# Comparing clinical features, severity and prognosis of autoimmune encephalitis and with and without oligoclonal bands

**DOI:** 10.3389/fneur.2023.1281276

**Published:** 2024-01-05

**Authors:** Hongfei Xue, Xiaochan Guo, Yushu Jiang, Lingzhi Qin, Xiaojuan Wang, Jiajia Xu, Shaomin Zuo, Qiuyan Liu, Wei Li

**Affiliations:** ^1^Department of Neurology, People’s Hospital of Zhengzhou University, Henan Provincial People’s Hospital, Zhengzhou, China; ^2^Department of Neonatology, Third Affiliated Hospital of Zhengzhou University, Zhengzhou, China; ^3^Department of Neurology, People’s Hospital of Henan University, Henan Provincial People’s Hospital, Zhengzhou, China

**Keywords:** autoimmune encephalitis, oligoclonal bands, the clinical assessment scale for autoimmune encephalitis, the modified Rankin scale, severity

## Abstract

**Objective:**

This study aimed to examine the clinical distinctions among patients diagnosed with autoimmune encephalitis (AE) based on the presence or absence of cerebrospinal fluid (CSF) oligoclonal bands (OCBs). Additionally, it sought to explore the relationship between OCBs and the severity and prognosis of autoimmune encephalitis.

**Methods:**

A retrospective analysis was conducted on 94 patients diagnosed with AE at the People’s Hospital of Zhengzhou University between October 2016 and June 2022. The patients were divided into OCB-positive and OCB-negative groups based on CSF-OCBs. Patient severity at admission was assessed utilizing the Clinical Assessment Scale for Autoimmune Encephalitis (CASE) and the modified Rankin scale (mRS). Additionally, some oligoclonal-positive patients underwent dynamic longitudinal analysis of cerebrospinal fluid test indices. The mRS score was ultimately employed to evaluate patients’ short-term prognosis (6 months) and long-term prognosis (at least 12 months) following immunotherapy.

**Results:**

Of the 94 patients, 34 (36.2%) belonged to the OCB-positive group, while 60 (63.8%) belonged to the OCB-negative group. The group with anti-n-methyl-d-aspartate receptor (anti-NMDAR) encephalitis exhibited the highest rate of oligoclonal positivity at 27 (49.1%), followed by anti-aminobutyric acid B receptor (GABABR) encephalitis with 4 cases (30.8%), anti-contactin-associated protein-like 2 (CASPR2) encephalitis with 2 cases (20%), and anti-leucine-rich glioma inactivating protein 1 (LGI1) encephalitis with 1 case (6.25%). No statistically significant differences were found between the two groups regarding gender, age, prodromal symptoms, psychiatric disorders, seizures, language disorders, motor dysfunction, cognitive dysfunction, tumor incidence, and magnetic resonance imaging (MRI) abnormalities (*p* > 0.05). The OCB-positive group exhibited higher rates of autonomic dysfunction, intensive care unit (ICU) admission, CSF leukocytes, and IgG index compared to the OCB-negative group (*p* < 0.05). Additionally, the OCB-positive group had significantly higher median CASE and mRS scores prior to immunotherapy than the OCB-negative group (*p* < 0.001 and *p* < 0.001). Furthermore, in both short-term follow-up and long-term follow-up, the OCB-positive group had a significantly lower proportion of patients with a favorable prognosis compared to the OCB-negative group (50% vs. 71.7, 61.8% vs. 83.3%; *p* = 0.036, *p* = 0.002).

**Conclusion:**

Autonomic dysfunction, ICU admission, leukocytes in the cerebrospinal fluid, and elevated IgG index are more commonly observed in OCB-positive patients. OCB-positivity has also been linked to the severity and prognosis of AE, making it a potential biomarker. Initial OCB testing aids clinicians in identifying potentially critically ill patients early and monitoring disease progression, thereby optimizing clinical treatment decisions.

## Introduction

Autoimmune encephalitis (AE) refers to a group of disorders where specific antibodies target intracellular proteins, synaptic receptors, ion channels, and/or neuronal surface proteins (1). Consequently, patients with AE exhibit various clinical manifestations, including seizures, psychiatric and behavior disorders, consciousness disorders, speech disorders, autonomic nervous dysfunction, cognitive dysfunction, and involuntary movement (2, 3). In severe cases, these symptoms can be life-threatening (2–4). Thus, early and aggressive treatment is crucial for improved functional outcomes and fewer relapses (4, 5). However, the diagnostic evaluation of relevant antibodies in the blood and cerebrospinal fluid may take several weeks, leading to a delay in diagnosis and immunotherapy initiation. Therefore, it is crucial to focus on classical routine cerebrospinal fluid (CSF) testing, which provides valuable indicators of the inflammatory process and aids in supporting the diagnosis and initiating early treatment (6, 7). Oligoclonal antibodies, a subclass of IgG, were initially discovered by Karcher et al. (8) in 1959 in patients with subacute sclerosing panencephalitis (SSPE). Subsequently, OCBs have been found in various immune-mediated or infectious neurological diseases, such as multiple sclerosis (MS), Lyme disease, neuro-syphilis, Behcet’s disease, and neuro-sarcoidosis (9–11). Especially in MS oligoclonal bands are biological markers of tremendous value (12). Prior studies have shown that CSF oligoclonal bands (OCBs) can be positive in AE, particularly in higher proportions among the anti-NMDAR encephalitis subtype (13, 14). Nevertheless, few studies have examined the correlation between OCBs and disease severity or the clinical prognosis of AE patients. Moreover, longitudinal analysis of the OCB state change is limited, and further research is needed to assess the prognostic value of CSF-OCB in the early stages of the disease and after immunotherapy.

Therefore, this study aimed to examine the clinical disparities between AE patients who tested positive or negative for CSF-OCB and the correlation between OCB and the severity and prognosis of AE.

## Materials and methods

### Ethical approval

This retrospective study received approval from the Ethics Committee of the People’s Hospital of Zhengzhou University. Additionally, it was conducted in adherence to the ethical standards outlined in the 1964 Declaration of Helsinki and its subsequent amendments. Furthermore, written informed consent was obtained from patients and proxies before they participated in the study.

### Study design and participants

This retrospective study enrolled patients diagnosed with probable AE and admitted to Zhengzhou University People’s Hospital between October 2016 and June 2022. A total of 188 patients were initially screened, excluding other potential differential diagnoses. All patients’ autoantibodies testing in both serum and CSF were performed through indirect immunofluorescence testing by third-party medical testing agencies. Only the patients who tested positive for autoantibodies against neuronal surface or synaptic proteins were included in the study. The antibody subtypes present comprised anti-n-methyl-d-aspartate receptor (NMDAR), anti-aminobutyric acid B receptor (GABABR), anti-leucine-rich glioma inactivating protein 1 (LGI1), and anti-contactin-associated protein-like 2 (CASPR2). Patients who had received immunotherapy before admission, those with incomplete clinical data, or those without OCB testing were excluded. The final analysis included 94 patients, all meeting the AE diagnostic criteria (15).

Paired serum and cerebrospinal fluid were tested for OCB using isoelectric focusing followed by IgG immunofixation during the recruited patients’ initial lumbar puncture (LP). In total, 184 OCB test results were collected from 94 patients. Longitudinal recordings were obtained from 16 anti-NMDAR patients who initially tested positive for OCB. If more than 5 LPs were performed, only LPs with changes in cell count, OCB status, or mRS score were reported.

### Data collection

The study gathered essential clinical data from patients, including demographic information (gender, age), the time interval between disease onset and initiation of immunotherapy, prodromal symptoms (fever, headache, respiratory symptoms, vomiting, diarrhea), clinical manifestations (mental behavior disorder, cognitive impairment, epileptic seizure, consciousness disorders, movement disorders, speech dysfunction, autonomic nervous dysfunction), presence of a coexisting tumor, ICU admission, and MRI results. Treatment data were collected for both first-line immunotherapy (corticosteroids, intravenous immunoglobulin, plasma exchange) and second-line immunotherapy (cyclophosphamide, rituximab, mycophenolate mofetil, tocilizumab, bortezomib, etc.) (15–18).

Following the initial LP upon admission, we gathered data on CSF white blood cell counts, total protein, albumin, and IgG levels. Additionally, we also collected serum albumin and IgG levels. From these collected results, we calculated *Q*_Alb_ and IgG index. Notably, *Q*_Alb_ represents the ratio of cerebrospinal fluid albumin to serum albumin (*Q*_Alb_ = Alb CSF/Alb serum). *Q*_Alb_ assesses the status of the blood-cerebrospinal fluid barrier. The upper limit of *Q*_Alb_ was calculated as 4 + (*α*/15) with *α* representing the patient’s age (19). Moreover, the IgG index provides insight into intrathecal immunoglobulin synthesis in the CSF.

### Scale assessment

The CASE and mRS scores were simultaneously evaluated upon admission (20, 21). Two independent neurologists (YJ and XW), blinded to the study’s objective, collaboratively reviewed the comprehensive charts, assessment scales, and discussed cases with discordant scores to reach a consensus. If an agreement could not be reached, a third senior neurologist (LQ) decided. The prognostic assessment was conducted for all patients through clinical or telephone follow-up at 6 months and at least 12 months after immunotherapy. In this study, mRS ≤2 was considered a good prognosis (4).

### Statistical analysis

SPSS IBM version 26.0 (IBM, Chicago, IL, United States) statistical software was used for statistical analysis. Data following normal distribution were presented as mean ± SD, while non-normally distributed data were reported as median (interquartile range, IQR). Categorical variables were analyzed with the chi-square test, and continuous variables were assessed using independent *t*-tests or the Mann–Whitney *U* test. *p* < 0.05 were defined as statistically significant.

## Results

### Clinical characteristics

**Figure 1:**
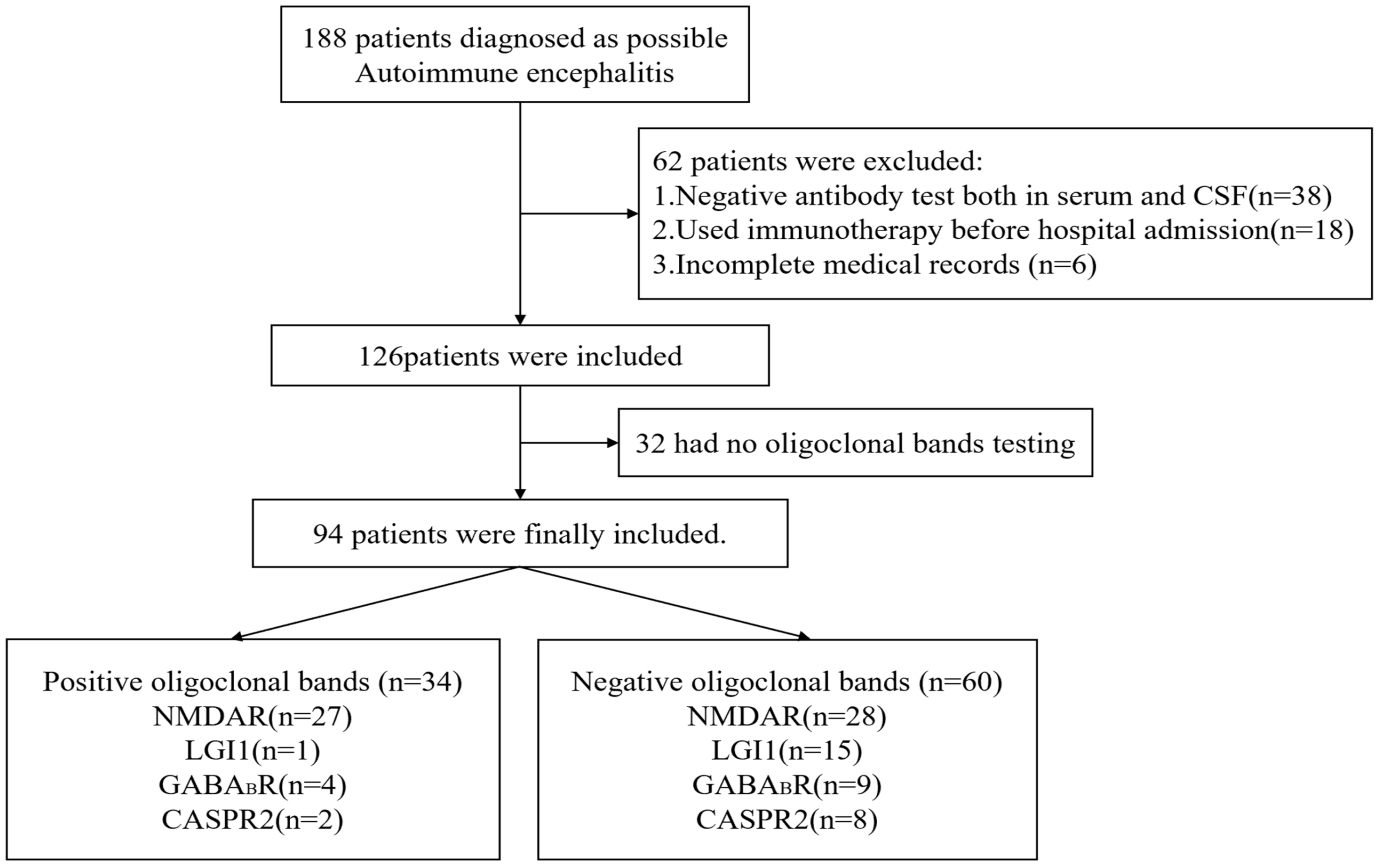
Flow chart of study patients.

Ninety-four patients with AE were included in this study, with 34 (36.2%) in the OCB-positive group and 60 (63.8%) in the OCB-negative group based on their OCB results. Anti-NMDAR encephalitis (49.1%) exhibited the highest rate of OCB-positivity, followed by GABABR encephalitis (30.8%), and CASPR2 encephalitis (20%). In contrast, LGI1 encephalitis demonstrated the lowest positivity rate (6.25%) (Figure 1). Table 1 provides a summary of the clinical characteristics of the enrolled patients. As shown, the median age was 33 years (IQR: 20–55 years) and female representation was 42.6%. Within the largest cohort of patients with anti-NMDAR encephalitis, the median age was 23 years (IQR: 17–32 years), and female representation was 56.4%.

**Table 1:** Demographics and clinical characteristics between OCB-positive and OCB-negative groups.

Variable		All (*n* = 94)	OCB− (*n* = 60)	OCB+ (*n* = 34)	*p*
Age (years, M, IQR)	AE	33 (20, 55)	44 (22, 58)	30 (20, 40)	0.070
	NMDAR	23 (17, 32)	22 (16, 27)	29 (18, 36)	0.281
Female, *n* (%)	AE	40 (42.6)	26 (43.3)	14 (41.2)	0.839
	NMDAR	31 (56.4)	19 (67.9)	12 (44.4)	0.080
Time from symptom onset until treatment, (days, M, IQR)	AE	17 (10, 31)	19 (10, 33)	17 (8, 23)	0.191
	NMDAR	12 (7, 18)	11 (7, 16)	14 (7, 20)	0.479
Prodromal symptoms, *n* (%)	AE	30 (31.9)	16 (26.7)	14 (41.2)	0.147
	NMDAR	24 (43.6)	12 (42.9)	12 (44.4)	0.906
Mental behavior disorder, *n* (%)	AE	51 (54.3)	29 (48.3)	22 (64.7)	0.126
	NMDAR	34 (61.8)	17 (60.7)	17 (63.0)	0.864
Epileptic seizure, *n* (%)	AE	58 (61.7)	35 (58.3)	23 (67.6)	0.372
	NMDAR	33 (60.0)	15 (53.6)	18 (66.7)	0.322
Consciousness disorders, *n* (%)	AE	26 (27.7)	13 (21.7)	13 (38.2)	0.084
	NMDAR	18 (32.7)	8 (28.6)	10 (37.0)	0.504
Cognitive impairment, *n* (%)	AE	44 (46.8)	26 (43.3)	18 (52.9)	0.370
	NMDAR	27 (41.9)	13 (46.4)	14 (51.9)	0.688
Movement disorders, *n* (%)	AE	22 (23.4)	12 (20.0)	10 (29.4)	0.300
	NMDAR	18 (32.7)	8 (28.6)	10 (37.0)	0.504
Speech dysfunction, *n* (%)	AE	13 (13.8)	6 (10.0)	7 (20.6)	0.153
	NMDAR	12 (21.8)	6 (21.4)	6 (22.2)	0.943
Autonomic nervous dysfunction, *n* (%)	AE	32 (34.0)	11 (18.3)	21 (61.8)	<0.001
	NMDAR	24 (43.6)	8 (28.6)	16 (59.3)	0.022
Tumor, *n* (%)	AE	10 (10.6)	7 (11.7)	3 (8.8)	0.935
	NMDAR	6 (10.9)	4 (14.3)	2 (7.4)	0.700
ICU admission, *n* (%)	AE	23 (24.5)	8 (13.3)	15 (44.1)	0.001
	NMDAR	16 (29.1)	4 (14.3)	12 (44.4)	0.030
Abnormal MRI, *n* (%)	AE	40 (42.6)	22 (36.7)	18 (52.9)	0.125
	NMDAR	27 (49.1)	14 (50.0)	13 (48.1)	0.891

M, median; IQR, interquartile range; NMDAR, NMDAR encephalitis subgroup; OCB−, negative oligoclonal bands; OCB+, positive oligoclonal bands; all of NMDAR encephalitis (*n* = 55); OCB-negative of NMDAR encephalitis (*n* = 28); OCB-positive of NMDAR encephalitis (*n* = 27).

Clinically, patients with AE had a median time of 17 days (IQR: 10–31 days) from symptom onset to treatment. Thirty (31.9%) patients presented with prodromal symptoms. Seizures (61.7%) were the most predominant clinical manifestation, followed by mental behavior disorder and cognitive impairment. Among the patients, 10 (10.6%) had underlying tumors, 23 (24.5%) required admission to the intensive care unit for severe conditions, and 40 (42.6%) exhibited abnormal brain MRI findings. In the total AE group, it was found that 11 (18.3%) patients in the OCB-negative group experienced autonomic dysfunction, whereas 21 (61.8%) patients in the OCB-positive group exhibited autonomic dysfunction (*p* < 0.001). In the OCB-negative group, 8 (13.3%) patients had ICU admission, while the OCB-positive group had 15 (44.1%) patients (*p* = 0.001). Furthermore, there was a discrepancy in autonomic dysfunction and ICU admission rates between the positive and negative groups within the anti-NMDAR encephalitis group (*p* < 0.05). No statistically significant differences were observed between the two groups regarding gender, age, prodromal symptoms, psychiatric disorders, seizures, language disorders, motor dysfunction, cognitive dysfunction, tumor incidence, and MRI abnormalities (*p* > 0.05).

**Figure 2:**
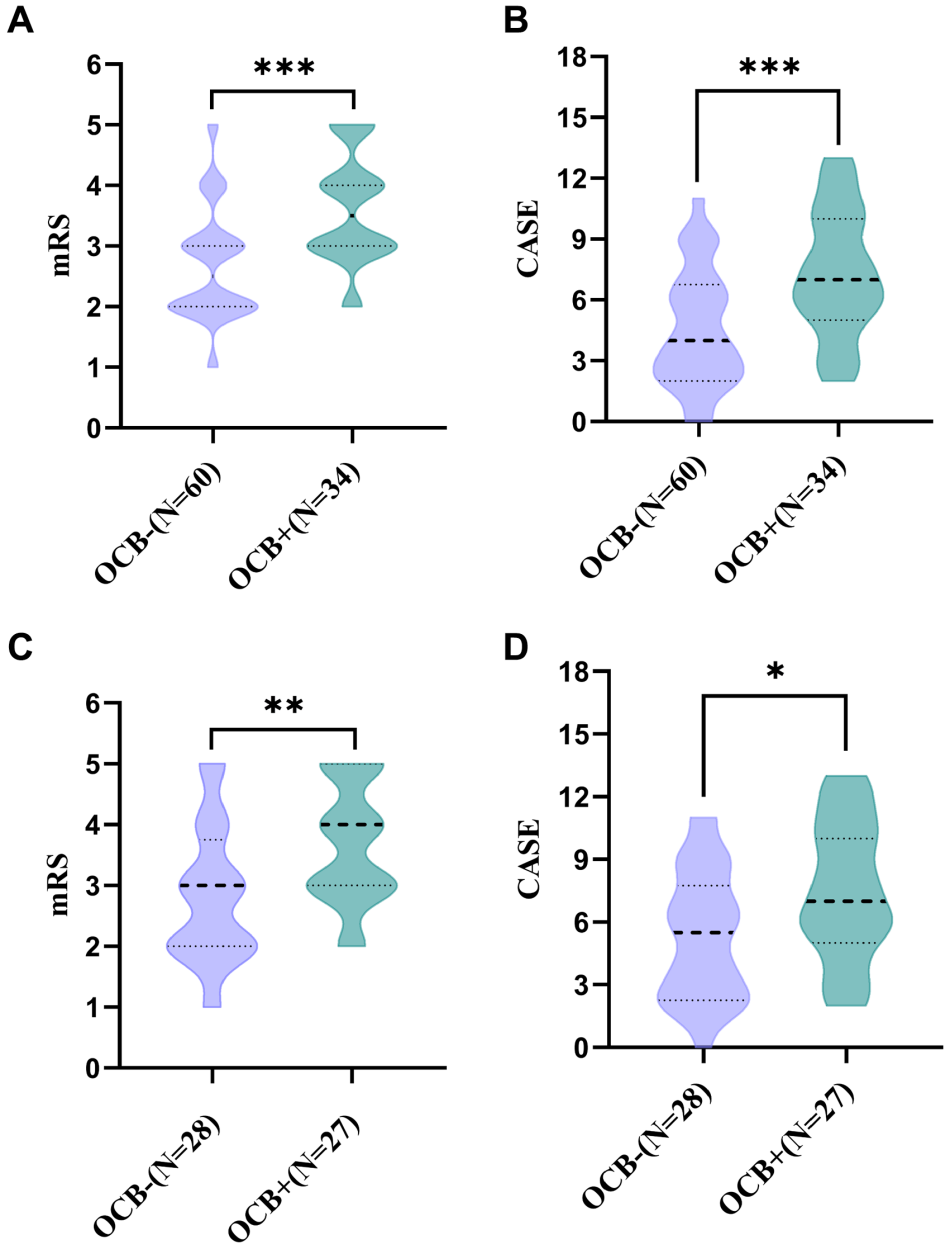
Comparison of mRS and CASE scores between OCB-negative and OCB-positive patients with autoimmune encephalitis at admission **(A,B)**. Comparison of mRS and CASE scores between OCB-negative and OCB-positive in anti-NMDAR encephalitis at admission **(C,D)**. ^*^p < 0.05, ^**^p < 0.01, and ^***^p < 0.001.

The disease severity of the patients at admission was evaluated using CASE and mRS scores. In the total AE group, the median mRS scores for the OCB-positive and OCB-negative groups were 3.5 (IQR: 3, 4) and 2.5 (IQR: 2, 3) (Figure 2A), respectively. Additionally, the median CASE scores for the OCB-positive and OCB-negative groups in the total AE group were 7 (IQR: 5, 10) and 4 (IQR: 2, 6.75) (Figure 2B). A significant difference in disease severity at admission between the two groups was observed for both mRS scores and CASE scores (*p* < 0.001 and *p* < 0.001) (Figures 2A,B). Additionally, differences in mRS and CASE scores were observed between the OCB-positive and OCB-negative groups in the anti-NMDAR encephalitis group (*p* < 0.01 and *p* < 0.05) (Figures 2C,D).

### CSF and serum findings at onset

The cerebrospinal fluid and serum blood test results are compared between the OCB-negative and OCB-positive groups in Table 2. The OCB-positive group exhibited a significantly greater increase in CSF leukocyte counts than the OCB-negative group (*p* < 0.001). The difference in CSF leukocyte counts was also statistically significant in the anti-NMDAR encephalitis group (*p* < 0.05). Furthermore, the OCB-positive group demonstrated a higher IgG index than the OCB-negative group in both the AE and anti-NMDAR encephalitis groups (*p* < 0.05). No significant differences were observed between the two groups regarding CSF protein, CSF albumin, CSF IgG, serum albumin, serum IgG and *Q*_Alb_ (*p* > 0.05).

**Table 2:** Comparison of the CSF and serum findings between OCB-positive and OCB-negative groups.

Variable		All (*n* = 94)	OCB− (*n* = 60)	OCB+ (*n* = 34)	*p*
CSF cell count (cell/μL, M, IQR)	AE	16 (4, 47)	9 (2, 23)	32 (17, 86)	<0.001
	NMDAR	21 (10, 75)	15 (8, 64)	40 (19, 105)	0.017
CSF protein (g/L, M, IQR)	AE	0.39 (0.27, 0.56)	0.40 (0.27, 0.54)	0.39 (0.26, 0.60)	0.774
	NMDAR	0.37 (0.26, 0.56)	0.36 (0.26, 0.45)	0.39 (0.28, 0.60)	0.235
CSF albumin (mg/L, M, IQR)	AE	184.00 (133.78, 244.43)	198.20 (146.73, 250.38)	163.00 (103.60, 244.43)	0.288
	NMDAR	177.10 (117.90, 243.60)	186.95 (115.15, 233.40)	169.10 (117.90, 246.90)	0.993
CSF IgG (mg/L, M, IQR)	AE	36.51 (27.27, 54.45)	35.46 (27.52, 48.12)	41.32 (26.30, 65.55)	0.269
	NMDAR	35.84 (27.62, 58.46)	35.37 (27.74, 49.35)	40.34 (26.62, 73.90)	0.350
Serum albumin (g/L, mean ± SD)	AE	39.04 ± 5.10	39.26 ± 5.09	38.69 ± 5.18	0.607
	NMDAR	40.07 ± 4.61	40.82 ± 4.86	39.29 ± 4.30	0.221
Serum IgG (g/L, M, IQR)	AE	10.60 (8.69, 13.55)	11.00 (8.52, 12.60)	10.42 (8.81, 14.78)	0.841
	NMDAR	10.56 (8.50, 15.21)	11.10 (8.38, 15.73)	10.29 (8.72, 14.48)	0.490
*Q*_Alb_ (M, IQR)	AE	4.76 (3.40, 6.43)	4.79 (3.82, 6.33)	4.45 (2.65, 6.85)	0.409
	NMDAR	4.35 (2.73, 6.42)	4.25 (2.62, 6.02)	4.36 (2.95, 6.94)	0.827
IgG index (M, IQR)	AE	0.68 (0.54, 0.88)	0.64 (0.50, 0.82)	0.78 (0.60, 1.13)	0.005
	NMDAR	0.75 (0.57, 1.05)	0.71 (0.53, 0.84)	0.85 (0.61, 1.31)	0.026

M, median; IQR, interquartile range; SD, standard deviation; NMDAR, NMDAR encephalitis subgroup; OCB−, negative oligoclonal bands; OCB+, positive oligoclonal bands; all of NMDAR encephalitis (*n* = 55); OCB-negative of NMDAR encephalitis (*n* = 28); OCB-positive of NMDAR encephalitis (*n* = 27).

### Serial CSF findings

**Figure 3:**
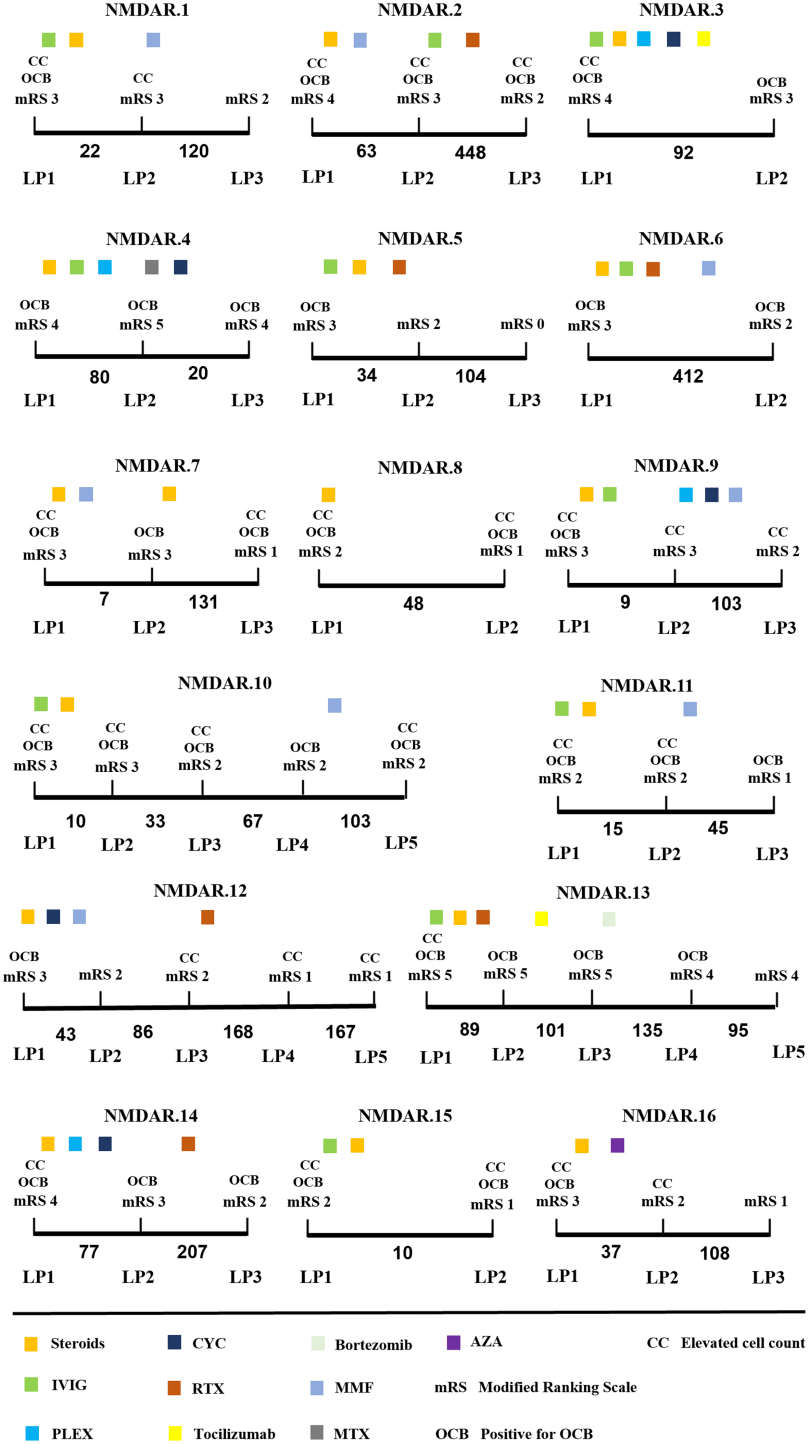
Longitudinal CSF findings and disease course of anti-NMDAR encephalitis patients. *x*-axis: time between LP in days. Steroids methylprednisolone; IVIG, intravenous immunoglobulins; PLEX, plasma exchange; CYC, cyclophosphamide; RTX, rituximab; MMF, mycophenolate mofetil; MTX, methotrexate; AZA, azathioprine.

Serial CSF analyses were conducted on 16 cases of OCB-positive anti-NMDAR encephalitis, encompassing 54 LPs (mean 3 LPs per patient). The median duration of the serial CSF analyses was 140 days (IQR:94–380 days). Overall, there was a progressive trend towards normalizing initial CSF pathology findings as time elapsed (Figure 3 and Table 3).

**Table 3:** Longitudinal CSF findings.

	LP1				LP2				LP3				LP4				LP5			
	CC	TP	*Q*_Alb_	OCB	CC	TP	*Q*_Alb_	OCB	CC	TP	*Q*_Alb_	OCB	CC	TP	*Q*_Alb_	OCB	CC	TP	*Q*_Alb_	OCB
NMDAR.1	19	0.38	3.76	+	6	0.11	2.06	−	2	0.52	6.75	−								
NMDAR.2	25	0.73	6.94	+	7	0.84	7.74	+	7	0.39	4.37	+								
NMDAR.3	18	0.19	2.17	+	2	0.17	1.74	+												
NMDAR.4	5	0.60	8.58	+	5	0.43	7.90	+	3	0.41	5.98	+								
NMDAR.5	3	0.24	1.10	+	1	0.10	1.04	−	1	0.10	1.69	−								
NMDAR.6	5	0.41	5.28	+	4	0.28	3.77	+												
NMDAR.7	41	0.32	4.88	+	5	0.21	3.32	+	12	0.47	5.12	+								
NMDAR.8	105	0.29	4.54	+	23	0.36	3.52	+												
NMDAR.9	23	0.10	2.35	+	7	0.26	3.63	−	9	0.42	6.35	−								
NMDAR.10	70	1.59	50.55	+	32	0.95	32.31	+	15	0.58	3.50	+	5	0.6	5.14	+	18	0.56	6.03	+
NMDAR.11	153	0.59	7.77	+	48	0.58	3.64	+	5	0.23	2.25	+								
NMDAR.12	4	0.60	7.39	+	2	0.54	7.14	−	7	0.76	6.88	−	14	0.71	6.58	−	8	0.31	3.63	−
NMDAR.13	6	0.47	3.91	+	5	0.25	4.05	+	4	0.45	7.33	+	2	0.20	2.12	+	3	0.30	4.54	−
NMDAR.14	7	0.56	5.10	+	1	0.45	4.89	+	2	0.33	6.11	+								
NMDAR.15	30	0.60	6.81	+	27	0.45	5.15	+												
NMDAR.16	20	0.51	3.37	+	6	0.23	5.59	−	4	0.21	4.09	−								
*p* ^*^																	0.001	0.030	0.234	

CC, cell count per μL; TP, total protein in g/L; *Q*_Alb_, Alb CSF/Alb serum; OCB, oligoclonal bands; p^*^, comparison of differential CSF cell counts, protein levels, and *Q*_Alb_ between initial and final patients. Normal values: CSF cell count (≤5/μL), CSF total protein (0.15–0.45 g/L).

During the initial CSF analysis, 12/16 patients exhibited elevated CSF cell counts, 8/16 patients had elevated cerebrospinal protein levels, and 6/16 patients showed impaired blood–brain barriers. During the final evaluation, there was a significant decrease in CSF cell counts and protein levels compared to the initial phase of the disease (*p* = 0.001 and *p* < 0.05) (Table 3). Additionally, only 2/16 patients showed impaired blood–brain barrier.

During the dynamic analysis of CSF, the OCB transitioned from positive to negative within a short period ranging from 9 to 43 days in 5 patients. Additionally, the OCB shifted from positive to negative after 420 days in 1 patient (NMDAR.13). All 6 patients received first-line therapy during the transition from positive to negative OCB status, with Steroids methylprednisolone combined with intravenous immunoglobulins being the most common. Interestingly, OCB status changed from positive to negative in two patients who applied Bortezomib (NMDAR.13) and azathioprine (NMDAR.16), respectively. During the final evaluation, 10 patients still exhibited OCB positivity, with a noteworthy mention of NMDAR.2 and NMDAR.6, both of whom maintained positive OCB status for over 1 year.

### Comparison of outcomes between OCB-positive and OCB-negative groups

**Figure 4:**
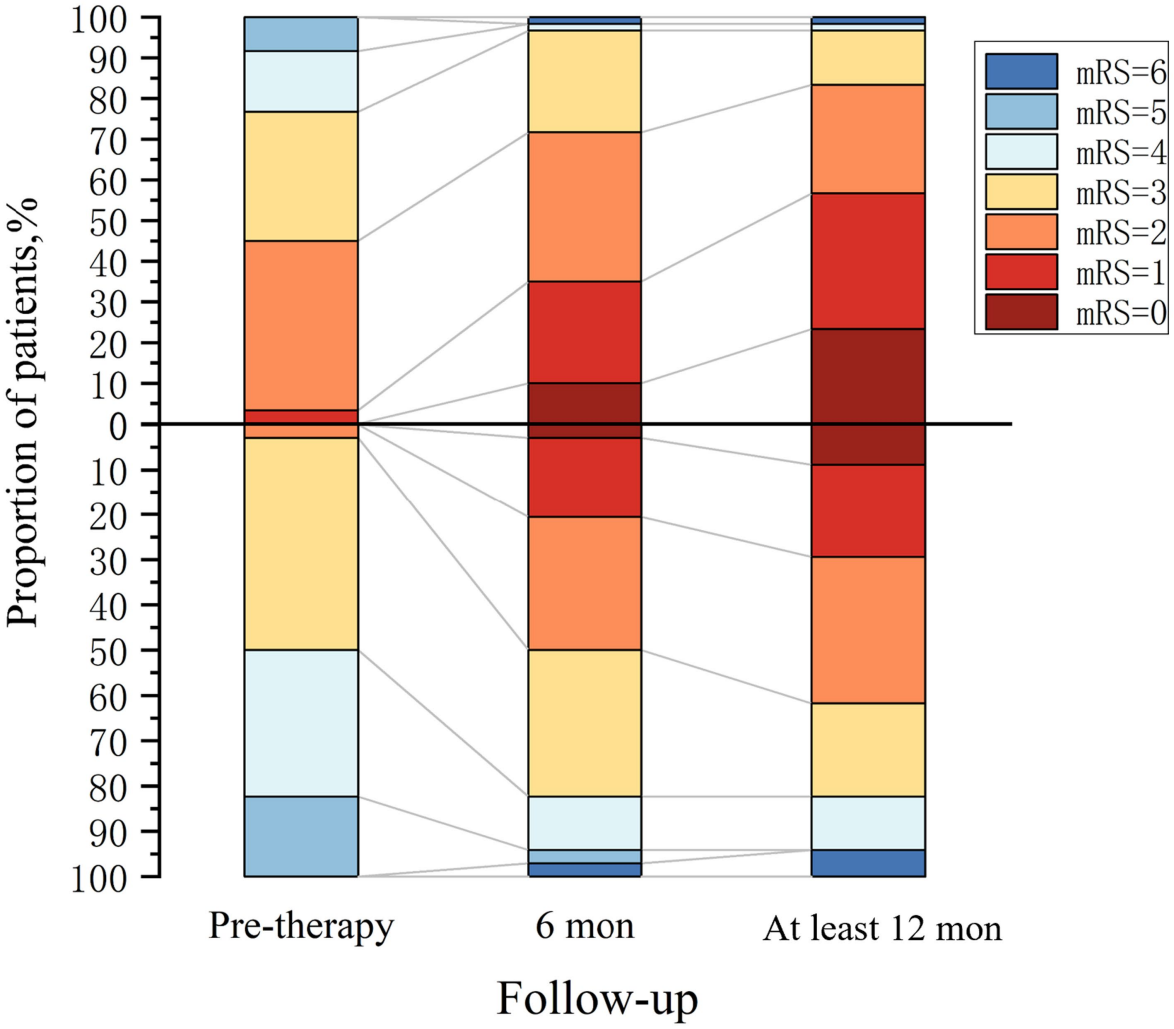
Clinical outcome in autoimmune encephalitis patients. OCB-negative group (above the *x*-axis, *n* = 60) and OCB-positive group (below the *x*-axis, *n* = 34).

All 94 patients were administered first-line immunotherapy. Among the patients in the OCB-negative group, 15% received second-line immunotherapy, while this percentage was 43.3% in the OCB-positive group. Figure 4 depicts the temporal changes in mRS scores for both groups. Following 6 months of immunotherapy, compared to patients in the 50% OCB-positive group, 71.7% of patients in the OCB-negative group became functionally independent (mRS ≤2), representing a significant difference (*p* = 0.036). During the long-term follow-up, conducted at a median time of 18 months (IQR: 14–35 months), it was found that 83.3% of patients in the OCB-negative group became functionally independent (mRS ≤2). In contrast, the OCB-positive group became functionally independent (mRS ≤2) in 61.8% of patients, representing a significant difference (*p* = 0.002).

## Discussion

Oligoclonal bands are a valuable clinical indicator for multiple sclerosis, OCB is detected in up to 95% of cases in MS (12, 22), and CSF-OCB’s diagnostic and prognostic applications have been well-established (23). Notably, OCB is part of the diagnostic criteria for anti-NMDAR encephalitis, allowing the diagnosis of possible anti-NMDAR encephalitis (15). Additionally, CSF-OCB is incorporated into the criteria for “autoantibody-negative but probable autoimmune encephalitis” (15). Thus, OCB testing is essential for accurate diagnosis and guiding appropriate therapeutic decisions, particularly in cases where antibodies are not detected. This further suggests that OCB may significantly impact the pathogenesis and progression of AE.

The oligoclonal positivity rate in our entire cohort of AE patients was 36.2%, with the highest rate observed in anti-NMDAR encephalitis at 49.1% and the lowest in the LGI1 encephalitis group at 6.25%. These rates align with previous reports of OCB-positivity ranging from 50% to 67% in anti-NMDAR encephalitis, while OCB-positivity in LGI1 encephalitis remains exceptionally rare at only 5% (7, 24). There were no significant differences in the incidence of major symptoms between the two groups, except for autonomic dysfunction and admission to the ICU. CSF pleocytosis, CSF-OCBs, and an elevated CSF IgG index are essential parameters in the diagnostic evaluation of AE. Importantly, CSF pleocytosis plays a central role in confirming the diagnosis of AE (15). Previous studies (6, 7) have also demonstrated that AE subtypes exhibiting frequent CSF pleocytosis often display frequent positivity for OCBs. Furthermore, our data revealed a significant disparity in pleocytosis and IgG index in the CSF between the OCB-positive and negative groups. Notably, these findings indicate the involvement of an IgG-induced immune response and the associated inflammation of the cerebrospinal fluid in the pathogenesis of AE (25).

A novel discovery is that patients with AE who tested positive for OCBs exhibited higher disease severity, as indicated by CASE and mRS scores upon admission. These two groups showed a significant disparity in disease severity at admission (*p* < 0.001 and *p* < 0.001). Likewise, the largest cohort of anti-NMDAR encephalitis patients yielded similar results. Thus, these findings suggest that CSF-OCB-positivity can serve as a risk factor for AE disease severity, aiding clinicians in monitoring disease progression and identifying patients at risk of developing severe symptoms at an early stage.

OCB is a valuable prognostic tool in predicting disease progression and clinical outcomes. Studies have demonstrated an elevated risk of developing MS in patients with suspected clinically isolated syndrome (CIS) when OCB is present (26, 27). Additionally, the absence of OCBs has been linked to improved prognosis and lower disability scores compared to patients with OCB-positive MS (28). In a small-scale study involving AE patients (*n* = 33) (6), the OCB status of individuals within the anti-NMDAR encephalitis subgroup (*n* = 7) was identified as a potential prognostic biomarker. Our findings indicated that the proportion of patients with a favorable prognosis was significantly lower in the OCB-positive group compared to the OCB-negative group during both short-term and long-term follow-up. Thus, these results suggest that OCB can potentially predict clinical prognosis in patients with AE.

We analyzed the CSF of a series of 16 patients with OCB-positive anti-NMDAR encephalitis. A trend towards the normalization of pathological findings, including elevated initial CSF cell counts, protein levels, and OCBs, was observed during the disease course. Previous research has noted that OCBs are transient in most anti-NMDAR encephalitis patients (6). Our study observed OCB conversion from positive to negative in only approximately one-third of the patients within a short period. From the last evaluation, 10 patients remained OCB-positive, and two maintained a positive OCB status for over 1 year. Longitudinal OCB status tracking time and different regional ethnic groups may account for the discrepancies. It has been suggested that there may be some inconsistencies in the OCB status of multiple sclerosis patients from ethnic groups at different latitudes (29). Nevertheless, it is indisputable that OCBs may persist for an extended period in anti-NMDAR encephalitis patients. In MS, CSF-OCBs are believed to originate from clonally expanded B cells within the intrathecal compartment (30). Evidence in anti-NMDAR encephalitis also suggests that intrathecal plasma cells can generate pathogenic anti-NMDAR antibodies (31). Additionally, postmortem investigations have revealed the presence of plasma cells/plasmablasts in the perivascular and interstitial spaces and infiltrating the brain parenchyma (32). Consequently, CSF-OCBs in anti-NMDAR encephalitis could be attributed to persistent and prolonged exposure to antigens within the central nervous system (CNS), triggering an ongoing immune response.

Evaluation based on mRS score confirms the significant impact of OCB conversion in patients with anti-NMDAR encephalitis, leading to stabilization or improvement in their condition. These findings are consistent with previous research (6). The distinctive immune profile within the CNS is associated with severe disease, indicating that intrathecal humoral immune responses may be crucial in determining the clinical course of anti-NMDAR encephalitis.

This study has several noted limitations. Firstly, this retrospective study was exclusively conducted in a single center, which introduces an inherent risk of bias. Secondly, due to the low prevalence of AE, the small sample size in subgroups other than the anti-NMDAR subgroup could not be analyzed with further precision. Thirdly, in the prognostic follow-up, it was not possible to obtain accurate CASE scores of patients in the short and long term by telephone follow-up due to the complexity of CASE scale items.

In summary, our study analyzed the relationship between clinical manifestations and CSF-OCBs in patients with AE. For the first time, a larger cohort explored the relationship between CSF-OCBs and the severity and prognosis of AE. Patients who tested positive for CSF-OCBs demonstrated a higher likelihood of experiencing autonomic dysfunction, requiring ICU admission, exhibiting CSF leukocytes, and having an elevated IgG index. Additionally, CSF-OCBs in AE are associated with disease severity and prognosis, suggesting its potential as a biomarker. Furthermore, initial testing of CSF-OCBs for monitoring disease progression and identifying potentially critically ill patients at an early stage is recommended, thereby optimizing clinical treatment decisions.

## Data availability statement

The raw data supporting the conclusions of this article will be made available by the authors, without undue reservation.

## Ethics statement

The studies involving humans were approved by the Medical Ethics Committee of the People’s Hospital of Zhengzhou University. The studies were conducted in accordance with the local legislation and institutional requirements. The participants provided their written informed consent to participate in this study.

## Author contributions

HX: Data curation, Formal analysis, Investigation, Writing – original draft. XG: Formal analysis, Investigation, Software, Writing – review & editing. YJ: Methodology, Supervision, Data curation, Writing – review & editing. LQ: Methodology, Supervision, Data curation, Writing – review & editing. XW: Methodology, Supervision, Data curation, Writing – review & editing. JX: Investigation, Data curation, Writing – review & editing. SZ: Investigation, Data curation, Writing – review & editing. QL: Investigation, Data curation, Writing – review & editing. WL: Funding acquisition, Methodology, Resources, Supervision, Writing – review & editing.
